# Supplementary material for: Modeling alpha-synuclein pathology in a human brain-chip to assess blood-brain barrier disruption
Source: Nat Commun. 2021 Oct 8;12:5907. doi: 10.1038/s41467-021-26066-5 (PMC8501050; doi:10.1038/s41467-021-26066-5)
Supplement: Supplementary file 1 — Supplementary Information [file 41467_2021_26066_MOESM1_ESM.pdf]

**Modeling Alpha-Synuclein Pathology in a Human Brain-Chip to Assess Blood-Brain Barrier Disruption**

**Pediaditakis et al.**

- **Supplementary Figures**
- **Supplementary Tables**

## Supplementary Figures

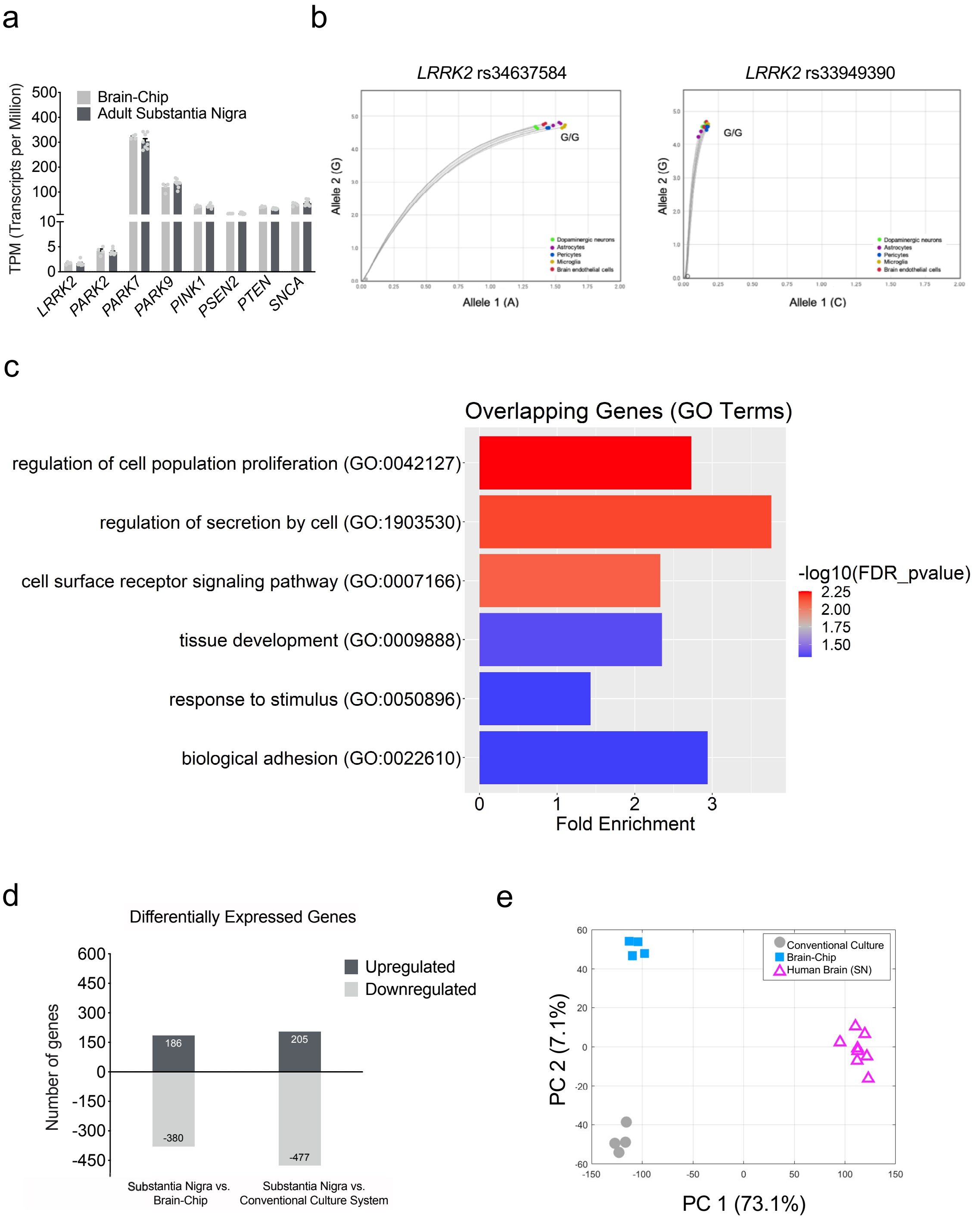


**Supplementary Figure 1.** Genetic similarities between the Brain-Chip and the normal adult substantia nigra. a) Average gene expression levels (Transcripts Per Million - TPM) of PD related genes in Brain-Chip and normal adult substantia nigra tissue. Sample sizes

were as follows: Substantia Nigra Brain-Chip, n=4 (independent chips) and adult substantia nigra, n=8 (independent biological specimens). Statistical analysis by two-way ANOVA with Tukey's multiple comparisons test (Not significant differences between groups). Error bars present mean±SEM. b) Allelic discrimination plots obtained for rs34637584 and rs33949390 using TaqMan genotyping assay. Dots represent genotypes and the squares on the bottom left of the plot are no-template control. The single nucleotide polymorphisms (SNPs) used for all the genotyping assays are listed in Supplementary Table 3. c) Substantia Nigra Brain-Chip exhibits higher transcriptomic similarity to adult substantia nigra than conventional cell culture. The results of the GO terms analysis using the 209 DE genes showed 6 significantly enriched (False Discovery Rate; FDR adjusted p-value<0.05) biological processes related to tissue development, response to a stimulus, biological adhesion, and cell surface receptor signaling pathway. The size of the bars indicates the fold-enrichment of the corresponding pathways. The p-values attained by the one-sided Fisher's exact test and controlled for low proportion of false positive using the FDR (False Discovery Rate) approach. d) Comparison of significantly up- or down-regulated genes between the following groups: i) Adult Substantia Nigra vs. Brain-Chip and ii) Adult Substantia Nigra vs. Conventional Cell Culture system (adjusted (adj) p-value<0.05 and |log2FoldChange|>1). For the differential gene expression analyses between the groups, the p-values attained by the two-sided Wald test and corrected for multiple testing using the Benjamini and Hochberg method. e) PCA generated using the RNA-seq data of cells from the conventional culture system (grey) (n=4), the Brain-Chip (blue) (n=4), and Adult SN (magenta) (n=8). A 2D-principal

component (PC) plot is shown with the first component along the X-axis and the second along the Y-axis. The proportion of explained variance is shown for each component.

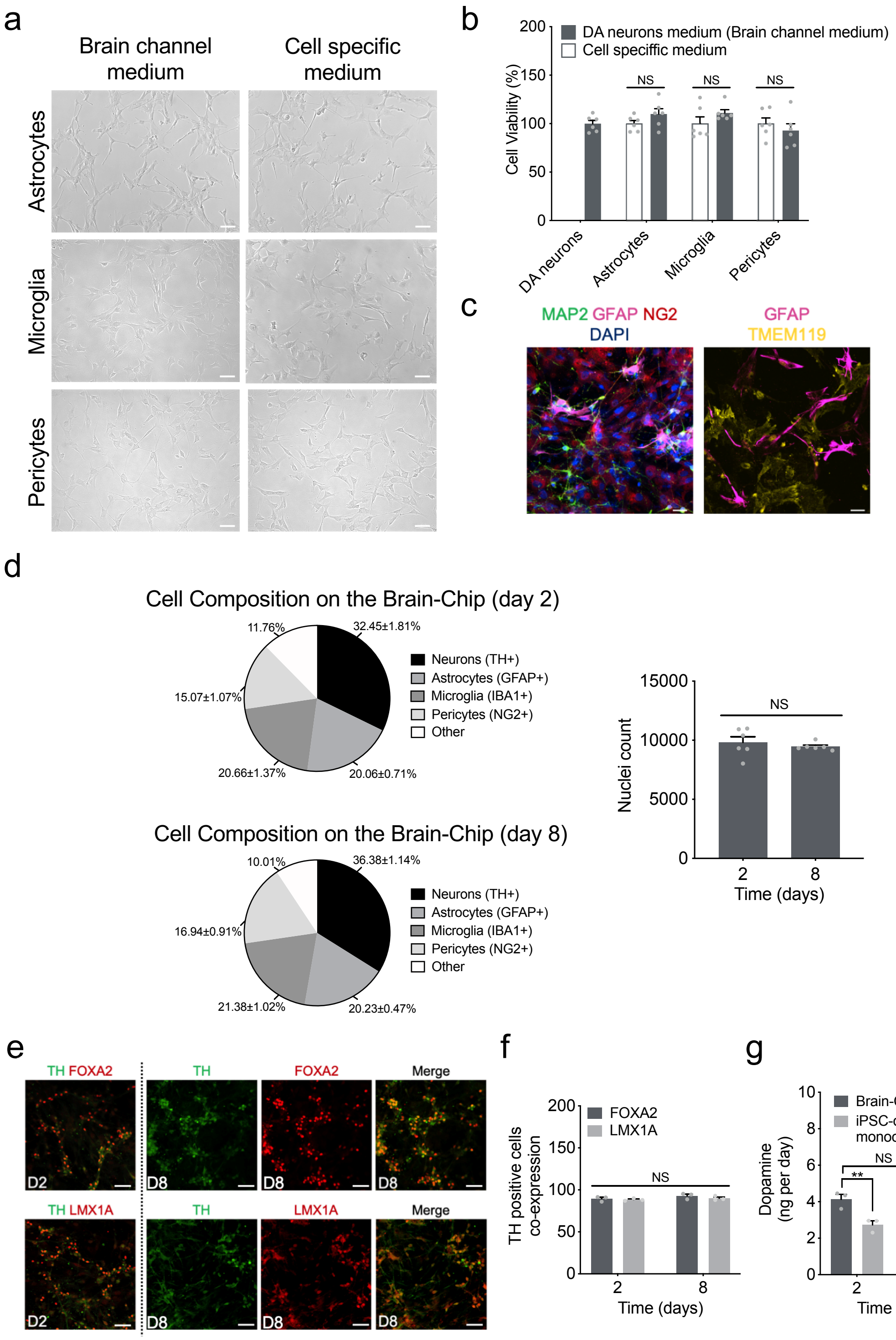


**Supplementary Figure 2.** Characterization of the human Brain-Chip. a) Morphology of astrocytes, microglia, and pericytes cultured in their respective culture medium or the "brain channel medium". Scale bars: 100 µm. b) Cellular metabolic activities assessed

by a (3-(4,5-dimethylthiazol-2-yl)-5-(3-carboxymethoxyphenyl)-2-(4-sulfophenyl)-2H-tetrazolium) MTS assay to compare astrocyte medium, microglia medium, pericyte medium, and brain channel medium (Dopaminergic neurons (DA) medium) (n=6/group, NS: Not Significant after comparison between brain channel medium versus each cell culture medium by two-sided unpaired t-test). Error bars present mean±SEM. c) Immunofluorescent microphotographs (left) validate the dopaminergic neurons with microtubule associated protein 2 (green, MAP2), astrocytes with glial fibrillary acidic protein (magenta, GFAP) and pericytes with neuron-glial antigen 2 (red, NG2), and DAPI (blue) for cell nuclei. Immunofluorescent microphotographs (right) of the glia culture: astrocytes (magenta, GFAP), and resting microglia (yellow, TMEM119). Scale bars: 50 µm. d) Relative percentage representation of each cell type in the brain channel throughout the length of culture (day 2, start and day 8, at the completion of the experiment). Staining with cell type specific markers (TH; dopaminergic neurons, GFAP; astrocytes, IBA1; microglia, NG2; pericytes) followed by normalization to the total cell number. Nuclei counts based on DAPI staining were similar in days 2 and 8. Statistical analysis is two-sided unpaired t-test (n=6 Brain-Chips; 8 randomly selected different areas per chip). Error bars represent mean±SEM. e) Double-label immunofluorescence with antibodies against tyrosine hydroxylase (green, TH), forkhead box A2 (red, FOXA2), and LIM homeobox transcription factor 1 alpha (red, LMX1A) at day 2 and 8. Scale bars: 100 µm. f) Percentage of FOXA2 and LMX1A positive neurons within the MAP2 and TH-positive population, in line with their floorplate midbrain phenotype (n=3 Brain-Chips with 3~5 randomly selected different fields of view per chip, NS: Not Significant). Statistical analysis is two-way ANOVA with Tukey's multiple comparisons test. Error bars represent

mean±SEM. g) ELISA for dopamine secreted into the medium of the brain channel and conventional iPSC-derived dopaminergic neuron monoculture on days 2 and 8. Statistical analysis is two-way ANOVA with Tukey's multiple comparisons test. (n=3 independent chips with duplicate technical replicates assayed per condition, Day 2: **P=0.0056, Day 8: **P=0.008, NS: Not Significant). Error bars represent mean±SEM.

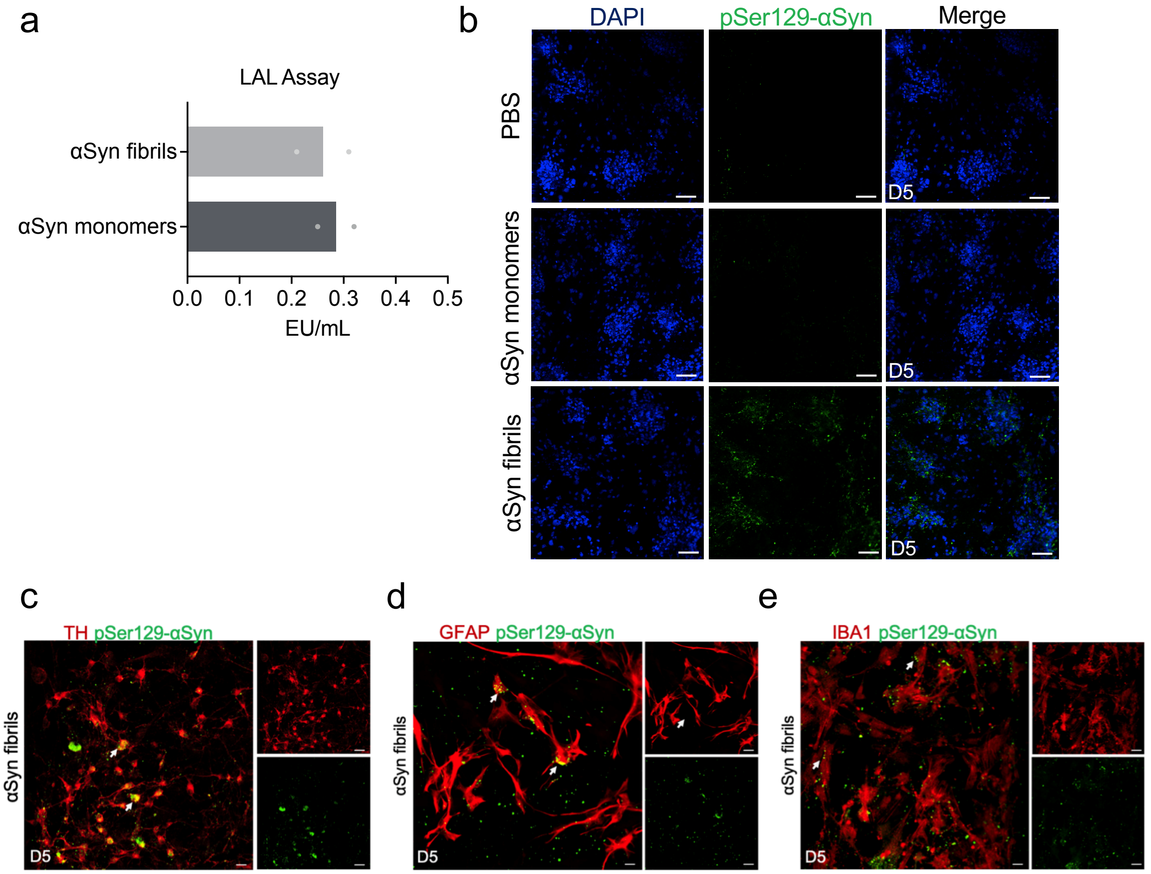


**Supplementary Figure 3.** Accumulation of phosphorylated αSyn in the αSyn fibril model**.** a) Detection of endotoxin levels using the Limulus amebocyte lysate (LAL) assay (n=2 measurements). b) Assessment of phosphorylated αSyn after three days post exposure to αSyn fibrils (D5 of the experiment). Immunofluorescence micrographs show the accumulation of phosphorylated αSyn (green, phospho-αSyn129 staining; blue, DAPI). Scale bars: 100 µm. c) Confocal images of double immunostaining for phospho-αSyn129 (green) and TH (red) in the brain channel after three days upon-exposure to αSyn fibrils (D5) (white arrows). Scale bars: 50 µm. d, e) αSyn fibrils are taken up by astrocytes and microglia after three days upon-exposure to αSyn fibrils (D5 of the experiment); double immunostaining for phospho-αSyn129 (green) and either GFAP (red) for astrocytes or IBA1 (red) for microglia (white arrows). Scale bars: 50 µm

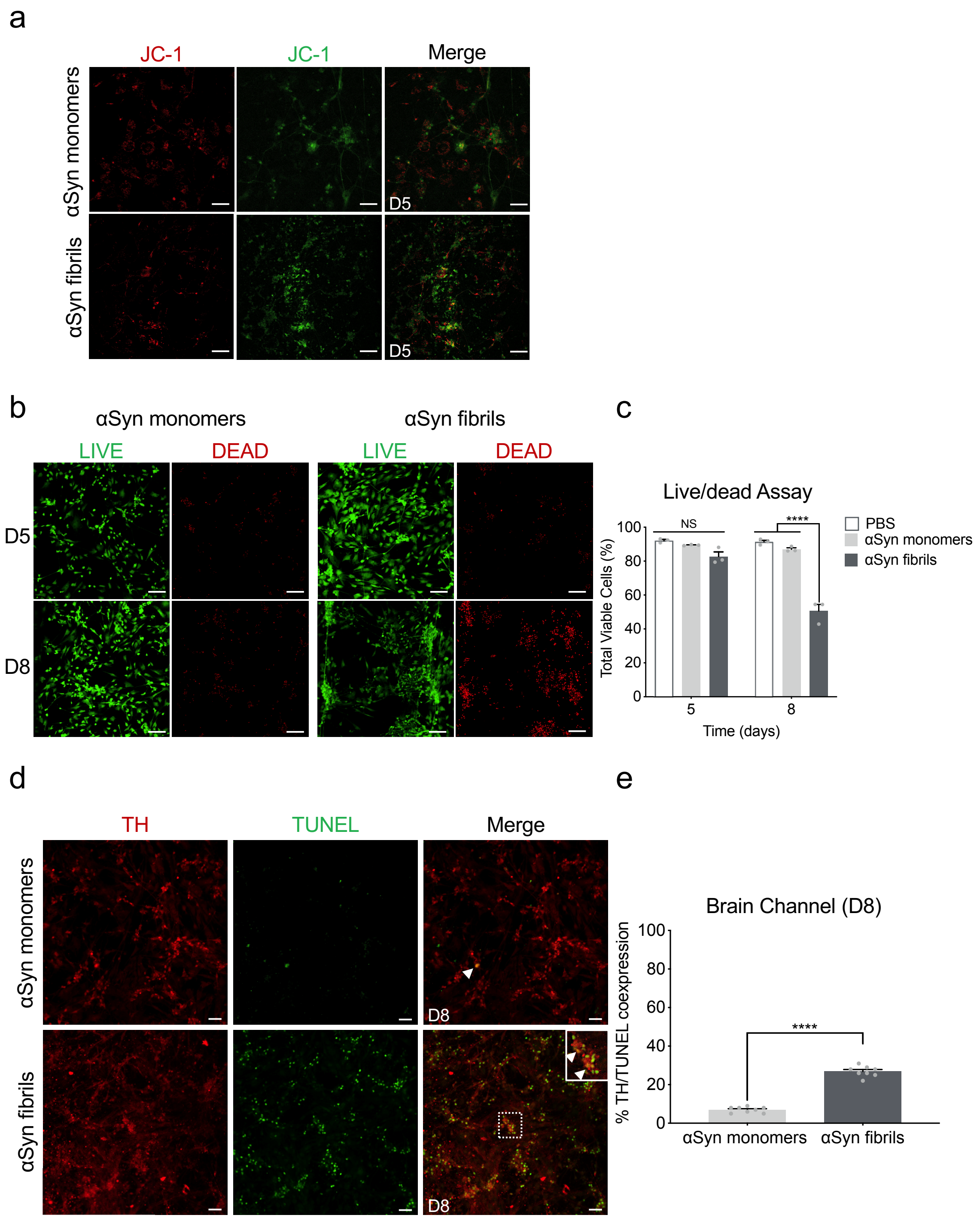


**Supplementary Figure 4.** Mitochondrial impairment and increased cell death in the αSyn fibril model. a) Effect of αSyn fibrils on the mitochondrial membrane potential after three days upon-exposure to αSyn fibrils (D5 of the experiment). Mitochondrial membrane

potential assessed by JC-1 staining on the brain side. Dual emission images (527 and 590nm) represent the signals from monomeric (green) and J-aggregate (red) JC-1 fluorescence. Scale bars: 100 µm. b) Cell viability (live/dead) assay following exposure to human αSyn fibrils. Live/Dead cell staining assay was designed to test the potential cytotoxicity of αSyn fibrils after three- and six-days upon-exposure to αSyn fibrils (D5 and D8 of the experiment, respectively). Scale bars: 100 µm. c) Data are expressed as the average live cells/total number of cells (sum of calcein AM positive and ethidium homodimer positive cells). Statistical analysis is two-way ANOVA with Tukey's multiple comparisons test (n=3 independent chips with 3~5 randomly selected different areas per chip, ****P<0.0001 compared to monomeric or PBS group, NS: Not Significant). Error bars present mean±SEM. d) Representative confocal images showing immunostaining for TH (red) and TUNEL (green) in the brain channel after six days upon-exposure to αSyn fibrils (D8 of the experiment) or αSyn monomers (white arrows). Scale bars: 100 µm. e) Percentage of the number of TUNEL positive within the TH-positive neurons. Statistical analysis is two-sided unpaired t-test (n=3 independent chips/3-4 distinct, randomly selected areas per chip, ****P<0.0001 compared to monomeric group). Error bars represent mean±SEM.

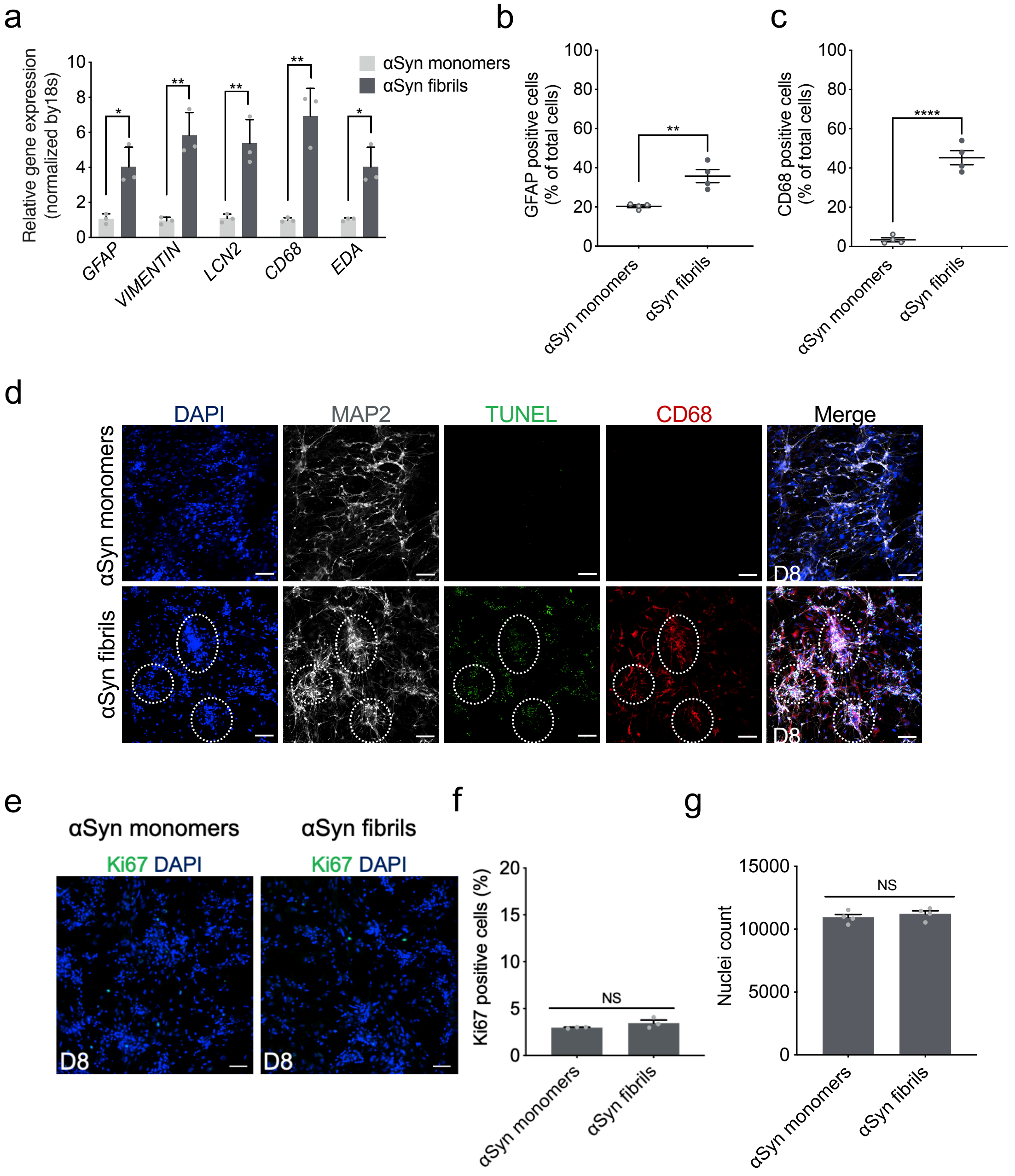


**Supplementary Figure 5.** αSyn fibrils-induced glial activation after six-days upon-exposure to αSyn fibrils (D8 of the experiment). a) Gene expression of specific markers for reactive astrocyte (*GFAP*, *VIMENTIN*, and *LCN2*) and activated microglia (*CD68* and *EDA*) in the brain channel at day six post exposure to αSyn fibrils or αSyn monomers. Statistical analysis is two-sided unpaired t-test (n=3 independent chips, *GFAP*:

*P=0.0376, *VIMENTIN*: **P=0.003, *LCN2*: **P=0.006, *CD68*: **P=0.0029, *EDA*: *P=0.0102 compared to αSyn monomeric group). Error bars present mean±SEM. b) Quantification of the number of GFAP-positive events per field of view normalized to the total number of cells. Statistical analysis is two-sided unpaired t-test (n=3 independent chips with 4 randomly selected different areas per chip, **P=0.004 compared to monomeric group). Error bars present mean±SEM. c) Quantification of the number of CD68-positive events per field of view normalized to the total number of cells. Statistical analysis is two-sided unpaired t-test (n=3 Brain-Chips with 4 randomly selected different areas per chip, ****P<0.0001 compared to the monomeric group). Error bars present mean±SEM. d) Representative confocal images showing immunostaining for MAP2 (grey), TUNEL (green), and CD68 (red) in the brain channel at day six post exposure to αSyn fibrils or αSyn monomers. Dashed circles show a large number of activated microglia that formed clusters around the apoptotic neurons. Scale bars: 100 μm. e) Immunocytochemical staining of proliferating Ki67-positive cells in αSyn monomeric or αSyn fibril-treated cultures. Scale bars: 100μm. f) Percentage of the Ki67-positive cells normalized to the total number of cells. Statistical analysis is two-sided unpaired t-test (4 randomly selected different areas per chip, n=3 independent chips/experimental group, NS: Not Significant). Error bars represent mean±SEM. Scale bars: 100μm. g) The nuclei count quantified from DAPI staining remained similar between the control and treated groups. Statistical analysis is two-sided unpaired t-test (n=3 Brain-Chips with 4 randomly selected different areas per chip, NS: Not Significant). Error bars represent mean±SEM.

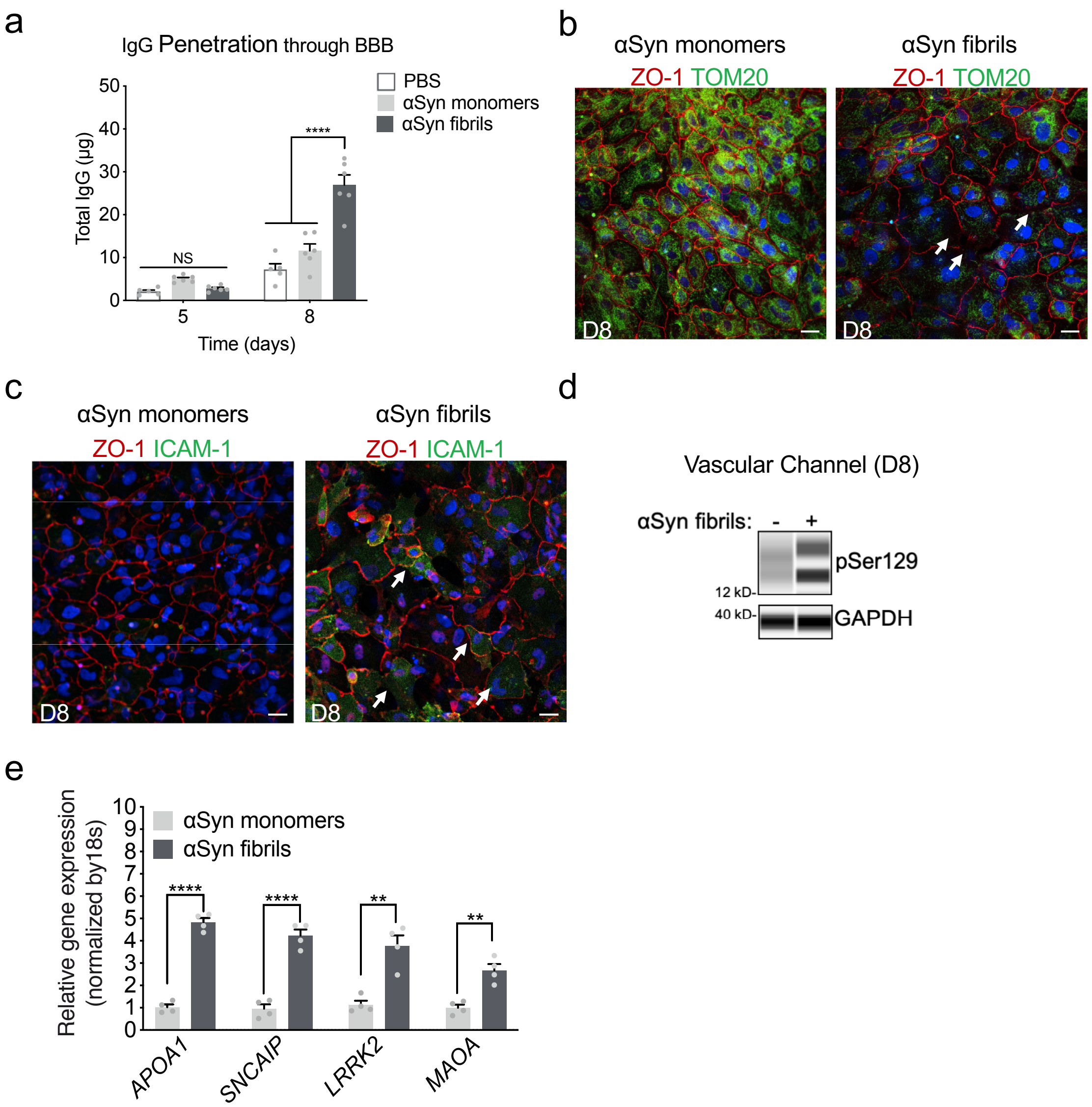


**Supplementary Figure 6.** Blood-Brain Barrier dysfunction in the αSyn fibril model**.** a) Quantitative barrier function analysis via permeability to immunoglobulin G (IgG1) at day three and six post exposure to αSyn fibrils, αSyn monomers or PBS. Statistical analysis is two-way ANOVA with Tukey's multiple comparisons test (n=5~8 independent chips, ****P<0.0001 compared to monomeric group, NS: Not Significant). Error bars present mean±SEM. b) Representative merged image of immunofluorescent staining of tight junction protein 1 (ZO-1, red), mitochondrial import receptor subunit (TOM20, green), and

cell nuclei (DAPI, blue) (white arrows). Scale bars: 100 µm. c) Representative merged image of immunofluorescent staining of tight junction protein 1 (ZO-1, red), intercellular adhesion molecule 1 (ICAM-1, green), and cell nuclei (DAPI, blue) (white arroes). Scale bars: 100 µm. d) Western blotting analysis of cell lysates from the vascular channel shows significant intracellular phosphorylation of αSyn at Ser129 (MW: 18kDa) following exposure to αSyn fibrils, whereas there was no effect upon exposure to PBS. For loading control, equal amounts of protein were immunoblotted with GAPDH antibody (MW: 37kDa). e) Expression of *APOA1*, *SNCAIP*, *LRRK2*, and *MAOA* in the vascular channel at day six post exposure to αSyn fibrils or αSyn monomers. Statistical analysis is two-sided unpaired t-test (n=4 independent chips, *APOA1* and *SNCAIP*: ****$P<0.0001$, *LRRK2*: **$P=0.019$, *MAOA*: **$P=0.0018$, compared to αSyn monomeric group). Error bars represent mean±SEM. All primers are listed in Supplementary Table 4.

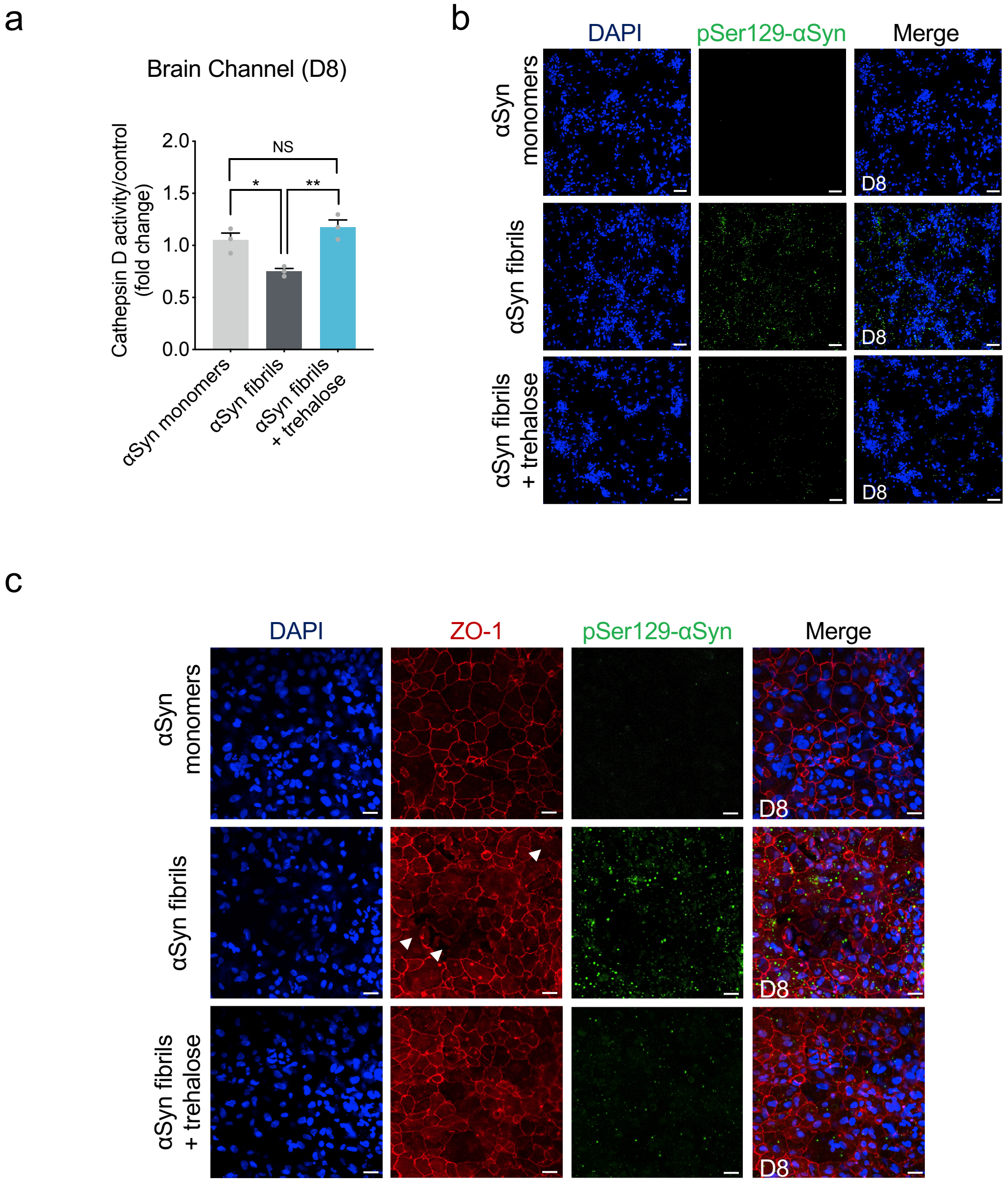


**Supplementary Figure 7.** Effect of trehalose on the accumulation of phosphorylated αSyn. a) Lysosomal cathepsin D activity at the brain channel on day 8. Statistical analysis by one-way ANOVA with Tukey's multiple comparisons test (n=3 independent chips with duplicate technical replicates assayed per condition, *P=0.0258, **P=0.005). Error bars represent mean±SEM. b) Immunofluorescence micrographs depict the accumulation of phosphorylated αSyn (green, phospho-αSyn129 staining; blue, DAPI) at day 8 in the αSyn

fibril model with or without trehalose treatment. Absence of pathology in the brain channel following exposure to monomer or co-treatment with fibrils and trehalose. c) Immunofluorescence micrographs depict the effect of fibrils on the accumulation of phosphorylated αSyn and tight junctions (green, phospho-αSyn129 staining; red, ZO-1; blue, DAPI) at day 8 in the αSyn fibril model with or without trehalose treatment. White arrows show the damaged endothelial junctions. Absence of pathology in the brain channel following exposure to monomer or co-treatment with fibrils and trehalose. Scale bars: 100 μm.

## Supplementary Tables

| Genes | adjusted P value (padj) |
|---|---|
| *TH* | 7.22E-11 |
| *PTX3* | 0.000587678 |
| *NURR1* | 0.319112246 |
| *GIRK2* | 0.097395788 |
| *FOXA2* | 0.000146612 |
| *LMX1A* | 0.001053186 |
| *GFAP* | 1.92E-170 |
| *AQP4* | 1.36E-36 |
| *GLAST* | 4.38E-36 |
| *ALDH1L1* | 0.0001441 |
| *TREM2* | 6.09E-05 |
| *TMEM119* | 1.19E-12 |
| *P2RY12* | 0.79861132 |
| *TH* | 7.22E-11 |
| *PTX3* | 0.000587678 |
| *NURR1* | 0.319112246 |

**Supplementary Table 1.** Adjusted P value per gene.

| Antibodies | Vendor | Cat. No. | Dilution |
|---|---|---|---|
| Rabbit anti-TH | abcam | ab6211 | 1:500 |
| Chicken anti-TH | abcam | ab76442 | 1:100 |
| Mouse anti-TH | Sigma | T2928 | 1:100 |
| Goat anti-GFAP | abcam | ab53554 | 1:300 |
| Rabbit anti-FOXA2 | Cell Signaling | 8186 | 1:200 |
| Rabbit anti-LMX1A | Sigma | ZRB1373 | 1:100 |
| Mouse ant-CD68 | abcam | ab955 | 1:100 |
| Mouse anti-ICAM-1 | R&D Systems | NET30 | 1:100 |
| Rabbit anti-MAP2 | abcam | ab32454 | 1:100 |
| Mouse anti-TOM20 | abcam | ab56783 | 1:100 |
| Rabbit anti-IBA1 | FCDI | 019-19741 | 1:50 |
| Mouse anti-phosphoSer129 | abcam | ab184674 | 1:100 |
| Rabbit anti-phosphoSer129 | Cell Signaling | 23706 | 1:100 |
| Rabbit anti-ki67 | abcam | ab197234 | 1:10 |
| Rabbit anti-CD68 | abcam | ab213363 | 1:100 |
| Rabbit anti-Cleaved Caspase-3 | abcam | ab32042 | 1:100 |
| Rabbit anti-NG2 | abcam | ab83178 | 1:100 |
| Rabbit anti-TMEM119 | abcam | ab185333 | 1:100 |
| Rabbit anti-pSer129-αSyn | abcam | ab51253 | 1:100 |
| Mouse anti-MAP2 | Thermo Fisher Scientific | MA512823 | 1:200 |
| Rabbit anti-CD31 | Thermo Fisher Scientific | RB-10333-P1 | 1:25 |
| Mouse anti-Claudin-1, | Thermo Fisher Scientific | 37-4900 | 1:25 |
| Mouse anti-Claudin-5 | Thermo Fisher Scientific | 35-2500 | 1:50 |
| Mouse anti-Occludin | Thermo Fisher Scientific | 33-1500 | 1:100 |
| Rabbit anti-ZO-1 | Thermo Fisher Scientific | 40-2200 | 1:200 |
| Mouse anti-ZO-1 | Thermo Fisher Scientific | ZO1-1A12 | 1:200 |
| GAPDH | RayBioteck Inc. | Not provided | - |
| Anti-Mouse Alexa Fluor® 488 | abcam | ab150105 | 1:300 |
| Anti-Goat Alexa Fluor® 647 | abcam | ab150135 | 1:300 |
| Anti-Rabbit Alexa Fluor® 568 | abcam | ab175470 | 1:300 |
| Anti-Rabbit Alexa Fluor® 488 | abcam | ab150073 | 1:300 |
| Anti-Mouse Alexa Fluor® 568 | abcam | ab175472 | 1:300 |
| Anti-Chicken Alexa Fluor® 568 | abcam | ab175477 | 1:300 |

**Supplementary Table 2.** List of antibodies.

| SNP ID | Gene Name | Location | Alleles | Context Sequence [VIC/FAM]: | TaqMan Assay ID # |
|---|---|---|---|---|---|
| rs34637584 | *LRRK2* | Chr.12: 40340400 on GRCh38 | G>A | CATCATTGCAAAGATTGCTGACTAC[A/G]GCATTGCTCAGTACTGCTGTAGAAT | C__63498123_10 |
| rs33949390 | *LRRK2* | Chr.12: 40320043 on GRCh38 | G>A,C,T | AAACACCCTAAGGGCATTATTTCGC[C/G]TAGAGATGTGGAAAAATTTCTTTCA | C__63497592_10 |

**Supplementary Table 3.** TaqMan genotyping assay IDs.

| Gene Symbol | Gene Name | TaqMan Assay ID # |
|---|---|---|
| *18s* | Eukaryotic 18S rRNA | Hs99999901_s1 |
| *GFAP* | Glial fibrillary acidic protein | Hs00909233_m1 |
| *VIMENTIN* | Vimentin | Hs00958111_m1 |
| *LCN2* | Lipocalin-2 | Hs01008571_m1 |
| *CD68* | CD68 molecule | Hs02836816_g1 |
| *EDA* | ectodysplasin A | Hs03025596_s1 |
| *APOA1* | apolipoprotein A1 | Hs00163641_m1 |
| *SNCAIP* | synuclein alpha interacting protein | Hs00917422_m1 |
| *LRRK2* | leucine rich repeat kinase 2 | Hs01115057_m1 |
| *MAOA* | monoamine oxidase A | Hs00165140_m1 |

**Supplementary Table 4.** Primers/probes information. The target genes were assessed using commercially available primers and probes.
