# Supplementary material for: Modeling alpha-synuclein pathology in a human brain-chip to assess blood-brain barrier disruption
Source: Nat Commun. 2021 Oct 8;12:5907. doi: 10.1038/s41467-021-26066-5 (PMC8501050; doi:10.1038/s41467-021-26066-5)
Supplement: Supplementary file 3 — Reporting Summary [file 41467_2021_26066_MOESM3_ESM.pdf]

nature research

Corresponding author(s): Iosif Pediaditakis
Katia Karalis

Last updated by author(s): Aug 12, 2020

Reporting Summary

Nature Research wishes to improve the reproducibility of the work that we publish. This form provides structure for consistency and transparency in reporting. For further information on Nature Research policies, see our Editorial Policies and the Editorial Policy Checklist.

Statistics

For all statistical analyses, confirm that the following items are present in the figure legend, table legend, main text, or Methods section.

n/a	Confirmed	
☐	☒	The exact sample size (*n*) for each experimental group/condition, given as a discrete number and unit of measurement
☐	☒	A statement on whether measurements were taken from distinct samples or whether the same sample was measured repeatedly
☐	☒	The statistical test(s) used AND whether they are one- or two-sided<br>*Only common tests should be described solely by name; describe more complex techniques in the Methods section.*
☒	☐	A description of all covariates tested
☐	☒	A description of any assumptions or corrections, such as tests of normality and adjustment for multiple comparisons
☐	☒	A full description of the statistical parameters including central tendency (e.g. means) or other basic estimates (e.g. regression coefficient) AND variation (e.g. standard deviation) or associated estimates of uncertainty (e.g. confidence intervals)
☐	☒	For null hypothesis testing, the test statistic (e.g. *F*, *t*, *r*) with confidence intervals, effect sizes, degrees of freedom and *P* value noted<br>*Give P values as exact values whenever suitable.*
☒	☐	For Bayesian analysis, information on the choice of priors and Markov chain Monte Carlo settings
☐	☒	For hierarchical and complex designs, identification of the appropriate level for tests and full reporting of outcomes
☒	☐	Estimates of effect sizes (e.g. Cohen's *d*, Pearson's *r*), indicating how they were calculated

Our web collection on statistics for biologists contains articles on many of the points above.

Software and code

Policy information about availability of computer code

Data collection	BioTek plate reader software: Synergy Neo GEN5 2.06<br>Zeiss Software: Zen 2.3 (black edition)<br>Olympus Software:CellSens Dimension 1.18<br>Opera Phenix (Perkin Elmer)<br>Transcriptomic data: Illumina HiSeq 2x150 system<br>QuantStudio 3 PCR System (Fisher Scientific)<br>RayBiotech's auto-western service
Data analysis	1. Statistical analysis-Microsoft Excel 365 and Prism 9<br>2. Confocal microscopy - Zen 2.3 (black edition) software<br>3. Harmony® imaging and analysis software 4.9<br>4. Densitometry - ImageJ software 1.52p<br>5. RNA-Sequencing - Illumina HiSeq 2x150 PE, Trimmomatic v.0.36, STAR aligner v.2.5.2b, Homo Sapiens GRCh38, Subread package v.1.5.2 featureCounts<br>6. Bioinformatics - RStudio Version 1.3.1056, packages: DEseq2 (BioConductor) v.1.30.1, ggplot2 v.3.3.3, matrixStats v.058.0, edgeR v.3.32.1, limma v3.46.0, MATLAB v.9.6.0.1072779 (R2019a), Statistics and Machine Learning Toolbox v.11.5

For manuscripts utilizing custom algorithms or software that are central to the research but not yet described in published literature, software must be made available to editors and reviewers. We strongly encourage code deposition in a community repository (e.g. GitHub). See the Nature Research guidelines for submitting code & software for further information.

Data

Policy information about availability of data

All manuscripts must include a data availability statement. This statement should provide the following information, where applicable:

- Accession codes, unique identifiers, or web links for publicly available datasets
- A list of figures that have associated raw data
- A description of any restrictions on data availability

In the Manuscript the following is stated:
Data Availability. All data generated or analyzed during this study are included in this published article (and its supplementary information files), and all additional information is available upon reasonable request to the authors. The microscope images have been deposited in Mendeley in a publicly available dataset (http://dx.doi.org/10.17632/bdnf2srwcg.1). In addition, all the uncropped WB images are provided in the source data file. RNA sequencing data have been deposited in the Gene Expression Omnibus (GEO) under the accession number GSE168870 (https://www.ncbi.nlm.nih.gov/geo/query/acc.cgi?acc=GSE168870). Source data are provided with this paper.

Field-specific reporting

Please select the one below that is the best fit for your research. If you are not sure, read the appropriate sections before making your selection.

- [x] Life sciences
- [ ] Behavioural & social sciences
- [ ] Ecological, evolutionary & environmental sciences

For a reference copy of the document with all sections, see nature.com/documents/nr-reporting-summary-flat.pdf

Life sciences study design

All studies must disclose on these points even when the disclosure is negative.

Sample size	Samples size was equal or larger than 3 in all experiments. The sample was chosen based on the evidence of previous work (Nature Communications, 10, 2621 (2019)), on which a sample size of n>2 was used per experimental group with and was sufficient on ensuring statistical power. No method was used to calculate the sample size.
Data exclusions	Data was not excluded from the experiment. According to company guidelines, failures might appeared due to following reasons:<br>1- Hardware failure (e.g. flow issues),<br>2- Contamination problems<br>However, none of these issues were apparent in the present study.
Replication	All experiments were replicated with at least 3 chip set-ups, and all attempts at replication were successful.
Randomization	Samples were allocated into experimental groups at random.
Blinding	The investigators were not blind to the design of the study due to different conditions and objectives per experiment, based on the experience of previous work (Nature Communications, 10, 2621 (2019)). However, the investigators were blinded to group allocation during experiments and data collection. Also, transcriptomics studies were carried out blinded by the operators.

Reporting for specific materials, systems and methods

We require information from authors about some types of materials, experimental systems and methods used in many studies. Here, indicate whether each material, system or method listed is relevant to your study. If you are not sure if a list item applies to your research, read the appropriate section before selecting a response.

Materials & experimental systems

n/a	Involved in the study	
[ ]	[x]	Antibodies
[ ]	[x]	Eukaryotic cell lines
[x]	[ ]	Palaeontology and archaeology
[x]	[ ]	Animals and other organisms
[x]	[ ]	Human research participants
[x]	[ ]	Clinical data
[x]	[ ]	Dual use research of concern

Methods

n/a	Involved in the study	
[x]	[ ]	ChIP-seq
[x]	[ ]	Flow cytometry
[x]	[ ]	MRI-based neuroimaging

Antibodies

Antibodies used	Rabbit anti-TH (abcam; ab6211)<br>Chicken anti-TH (abcam; ab76442)

Goat anti-GFAP (abcam; ab53554)
Mouse anti-TH (Sigma; T2928)
Rabbit anti-FOXA2 (Cell Signaling Tech.;8186)
Rabbit anti-LMX1A (Sigma; ZRB1373)
Mouse ant-CD68 (abcam; ab955)
Mouse anti-ICAM-1 (R&D Systems; NET30)
Rabbit MAP2 (abcam; ab32454)
Mouse anti-TOM20 (abcam; ab56783)
Rabbit anti-IBA1 (FCDI; 019-19741)
Mouse anti-phosphoSer129 (abcam; ab184674)
Rabbit anti-phosphoSer129 (Cell Signaling Tech.; 23706)
Rabbit anti-ki67 (abcam; ab197234)
Rabbit anti-CD68 (abcam; ab213363)
Rabbit ant- Cleaved Caspase-3 (abcam; ab32042)
Rabbit anti-NG2 (abcam; ab83178)
Rabbit anti-TMEM119 (abcam; ab185333)
Rabbit anti-pSer129-αSyn (abcam; ab51253)
Mouse anti-MAP2 (Thermofisher Scientific; MA512823)
Rabbit anti-CD31; (Thermo Fisher Scientific; RB-10333-P1)
Mouse anti-Claudin-1, (Thermo Fisher Scientific; 37-4900)
Mouse anti-Claudin-5 (Thermo Fisher Scientific; 35-2500)
Mouse anti-Occludin (Thermo Fisher Scientific; 33-1500)
Rabbit anti-ZO-1 (Thermo Fisher Scientific; 40-2200)
Mouse anti-ZO-1 (Thermo Fisher Scientific; (ZO1-1A12)
Donkey Anti-Mouse IgG H&L (Alexa Fluor® 488) (abcam; ab150105)
Donkey polyclonal Secondary Antibody to Goat IgG - H&L (Alexa Fluor® 647) (abcam; ab150135)
Donkey Anti-Rabbit IgG H&L (Alexa Fluor® 568) (abcam; ab175470)
Donkey polyclonal Secondary Antibody to Rabbit IgG - H&L (Alexa Fluor® 488) (abcam; ab150073)
Donkey Anti-Mouse IgG H&L (Alexa Fluor® 568) (abcam; ab175472)
Goat Anti-Chicken IgY H&L (Alexa Fluor® 568) (abcam; ab175477)

Validation

Only company-validated antibodies were used in this work for immunocytochemistry analyses. All Cell Signaling antibodies were validated by the manufacturer using various approaches (https://www.cellsignal.com/about-us/our- approach-process/cst-antibody-validation-principles). All Abcam antibodies used in this report, were validated by the manufacturer as well (https://www.abcam.com/primary-antibodies/how-we-validate-our-antibodies). All Thermo Fisher Scientific antibodies were validated by the manufacturer using various approaches (https://www.thermofisher.com/us/en/home/life-science/antibodies/invitrogen-antibody-validation).
Specifically, the applications of the antibodies listed in this report can be found below:

TH (abcam; ab6211) is a rabbit polyclonal antibody developed for Tyrosine Hydroxylase. It has been validated for use in immunohistochemistry, immunocytochemistry, and western blot of mouse or rats. https://www.abcam.com/tyrosine-hydroxylase-antibody-ab6211.html
TH (abcam; ab76442) is a chicken polyclonal antibody to human Tyrosine Hydroxylase. It has been validated for use in immunohistochemistry of only mouse species. https://www.abcam.com/tyrosine-hydroxylase-antibody-ab76442.html
TH (Sigma; T2928) is a mouse monoclonal antibody developed for Tyrosine Hydroxylase. It has applications in immunohistochemistry, immunoprecipitation, microarray, and western blot of guinea pig, bovine, sheep, monkey, rat, human, rabbit species. https://www.sigmaaldrich.com/US/en/product/sigma/t2928
GFAP (abcam; ab53554) is a goat polyclonal antibody developed for GFAP. It has been validated for only western blots of mouse, rat, or human species. https://www.abcam.com/gfap-antibody-ab53554.html
FOXA2 (Cell Signaling Tech.;8186) is a rabbit monoclonal antibody for FoxA2/HNF3β. Its applications include western blot, immunoprecipitation, and immunocytochemistry of human, mouse, and rat models. https://www.cellsignal.com/products/primary-antibodies/foxa2-hnf3b-d56d6-xp-rabbit-mab/8186
LMX1A (Sigma; ZRB1373) is a rabbit monoclonal antibody for LMX-1 expressed in HEK 293 cells. It has been validated for use in immunocytochemistry, immunohistochemistry, and western blots of humans or hamsters. https://www.sigmaaldrich.com/US/en/product/sigma/zrb1373
CD68 (abcam; ab955) is a mouse monoclonal antibody to CD68. It has been validated for use in western blot, immunohistochemistry, immunocytochemistry of human, mouse, rabbit, and rat. https://www.abcam.com/cd68-antibody-kp1-ab955.html
ICAM-1 (R&D Systems; NET30) is a mouse monoclonal antibody that detects human ICAM-1. It has been validated for use in western blot and immunocytochemistry for humans and hamsters. https://www.rndsystems.com/products/human-icam-1-cd54-antibody-14c11_mab720
MAP2 (abcam; ab32454) is a rabbit polyclonal antibody used to detect MAP-2. It is suitable for immunohistochemistry, immunocytochemistry, and western blot of mouse, rat, and human species. https://www.abcam.com/map2-antibody-neuronal-marker-ab32454.html
TOM20 (abcam; ab56783) is a mouse monoclonal antibody to human TOM20. It is suitable for western blot, immunohistochemistry, flow cytometry, immunocytochemistry, and immunoprecipitation for mouse, rat, and human species.
IBA1 (FCDI; 019-19741) is a rabbit polyclonal antibody for ionized calcium binding adaptor molecule 1 (IBA1) of human microglia. It has been validated for immunocytochemical staining for human, mouse, and rat species. https://www.fujifilmcdi.com/anti-iba1-polyclonal-antibody-019-19741
phosphoSer129 (abcam; ab184674) is a mouse monoclonal antibody to human Phospho-α-Synuclein (Ser129). It has been validated for immunohistochemistry of only human species. https://www.abcam.com/alpha-synuclein-phospho-s129-antibody-p-syn81a-

ab184674.html
phosphoSer129 (Cell Signaling Tech.; 23706) is a rabbit monoclonal antibody to Phospho-α-Synuclein (Ser129). Its applications include western blot, immunoprecipitation, immunohistochemistry, chromatin immunoprecipitation, immunofluorescence, flow cytometry, and lastly as an ELISA-peptide. Its cross reactive for human, mouse, rat, hamster, monkey, chicken, dog, pig, horse, and virus. https://www.cellsignal.com/products/primary-antibodies/phospho-a-synuclein-ser129-d1r1r-rabbit-mab/23706
ki67 (abcam; ab197234) is a rabbit monoclonal antibody to KI67. It has been validated for use in flow cytometry and immunocytochemistry of only human species. https://www.abcam.com/alexa-fluor-488-ki67-antibody-epr3610-ab197234.html
CD68 (abcam; ab213363) is a rabbit monoclonal antibody to CD68. It is suitable for western blot, immunohistochemistry, and immunocytochemistry for only humans. https://www.abcam.com/cd68-antibody-epr20545-ab213363.html
Cleaved Caspase-3 (abcam; ab32042 is a rabbit monoclonal antibody to human Cleaved-Caspase-3. It has been validated for western blotting and immunocytochemistry for only humans. https://www.abcam.com/cleaved-caspase-3-antibody-e83-77-ab32042.html
NG2 (abcam; ab83178) is a rabbit polyclonal antibody for Chondroitin sulfate proteoglycan NG2. Its applications include immunocytochemistry, immunohistochemistry, and western blots for both humans and rats. https://www.citeab.com/antibodies/769446-ab83178-anti-ng2-antibody
TMEM119 (abcam; ab185333) is a rabbit polyclonal antibody for human TMEM119 – C-terminal. It has been tested for immunohistochemistry for only human species. https://www.abcam.com/tmem119-antibody-c-terminal-ab185333.html
pSer129-αSyn (abcam; ab51253) is a rabbit monoclonal antibody to human Alpha-synuclein (phospho S129). It is suitable for immunohistochemistry, western blot, and ELISA for mouse, rat, and humans. https://www.abcam.com/alpha-synuclein-phospho-s129-antibody-ep1536y-ab51253.html
MAP2 (Thermofisher Scientific; MA512823) is a mouse monoclonal antibody for brain microtubule associated protein 2 (MAP-2). It has been validated for use in western blots, immunohistochemistry, and immunocytochemistry for human, mouse, and rat species. https://www.thermofisher.com/antibody/product/MAP2-Antibody-clone-AP20-Monoclonal/MA5-12823
CD31 (Thermo Fisher Scientific; RB-10333-P1) is a rabbit polyclonal antibody for CD31/PECAM-1 of endothelial cells. It has applications in immunohistochemistry stainings for human species. https://www.fishersci.com/shop/products/lab-vision-cd31-pecam-1-endothelial-cell-marker-rabbit-polyclonal-antibody-bsa-azide/RB10333P1
Claudin-1, (Thermo Fisher Scientific; 37-4900) is a mouse monoclonal antibody for human Claudin-1. It has been validated for use in western blots, immunohistochemistry, immunocytochemistry, flow cytometry, ELISA, and immunoprecipitation of dog, human, mouse, and rat species. https://www.thermofisher.com/antibody/product/Claudin-1-Antibody-clone-2H10D10-Monoclonal/37-4900
Claudin-5 (Thermo Fisher Scientific; 35-2500) is a mouse monoclonal antibody for Claudin-5. It has been validated for use in western blots, immunohistochemistry, immunocytochemistry, flow cytometry, ELISA, and immunoprecipitation for mouse, rat, and human species. https://www.thermofisher.com/antibody/product/Claudin-5-Antibody-clone-4C3C2-Monoclonal/35-2500
Occludin (Thermo Fisher Scientific; 33-1500) is a mouse monoclonal antibody for human Occludin. It has been validated for use in western blots, immunohistochemistry, immunocytochemistry, flow cytometry, ELISA, and immunoprecipitation for dog, mouse, rat, and human species. https://www.thermofisher.com/antibody/product/Occludin-Antibody-clone-OC-3F10-Monoclonal/33-1500
ZO-1 (Thermo Fisher Scientific; 40-2200) is a rabbit polyclonal antibody for the ZO-1 protein. It has been validated for use in western blots, immunohistochemistry, immunocytochemistry, and immunoprecipitation for dog, mouse, rat, and human species. https://www.thermofisher.com/antibody/product/ZO-1-Antibody-Polyclonal/40-2200
ZO-1 (Thermo Fisher Scientific; (ZO1-1A12) is a mouse monoclonal antibody for the human recombinant ZO-1 fusion protein. It has been validated for use in western blots, immunohistochemistry, immunocytochemistry, flow cytometry, ELISA, and immunoprecipitation for mouse, rhesus monkey, and human species. https://www.thermofisher.com/antibody/product/ZO-1-Antibody-clone-ZO1-1A12-Monoclonal/33-9100
Donkey Anti-Mouse IgG H&L (Alexa Fluor® 488) (abcam; ab150105) is a donkey secondary antibody for conjugation at Alexa Fluor® 488 (Ex: 495nm, Em: 519nm.) It is suitable for immunohistochemistry, immunocytochemistry, ELISA, and flow cytometry for only the mouse species. https://www.abcam.com/donkey-mouse-igg-hl-alexa-fluor-488-ab150105.html
Donkey polyclonal Secondary Antibody to Goat IgG - H&L (Alexa Fluor® 647) (abcam; ab150135) is a donkey polyclonal secondary antibody for conjugation at Alexa Fluor® 647 (Ex: 652nm, Em: 668nm.) It has been validated for immunohistochemistry, immunocytochemistry, ELISA, and flow cytometry for only the goat species. https://www.abcam.com/donkey-goat-igg-hl-alexa-fluor-647-preadsorbed-ab150135.html
Donkey Anti-Rabbit IgG H&L (Alexa Fluor® 568) (abcam; ab175470) is a donkey secondary antibody for conjugation at Alexa Fluor® 568 (Ex: 578nm, Em: 603nm). Its applications are for immunohistochemistry, immunocytochemistry, ELISA, and flow cytometry for only the rabbit species. https://www.abcam.com/donkey-rabbit-igg-hl-alexa-fluor-568-ab175470.html
Donkey polyclonal Secondary Antibody to Rabbit IgG - H&L (Alexa Fluor® 488) (abcam; ab150073) is a donkey polyclonal secondary antibody for conjugation at Alexa Fluor® 488. (Ex: 495nm, Em: 519nm.) It is suitable for immunohistochemistry, immunocytochemistry, ELISA, and flow cytometry for only the rabbit species. https://www.abcam.com/donkey-rabbit-igg-hl-alexa-fluor-488-ab150073.html
Donkey Anti-Mouse IgG H&L (Alexa Fluor® 568) (abcam; ab175472) is a donkey secondary antibody for conjugation at Alexa Fluor® 568 (Ex: 578nm, Em: 603nm.) It is suitable for immunohistochemistry, immunocytochemistry, ELISA, and flow cytometry for only the mouse species. https://www.abcam.com/donkey-mouse-igg-hl-alexa-fluor-568-ab175472.html
Goat Anti-Chicken IgY H&L (Alexa Fluor® 568) (abcam; ab175477) is a goat secondary antibody for conjugation at Alexa Fluor® 568 (Ex: 578nm, Em: 603nm). It has been validated for immunohistochemistry, immunocytochemistry, ELISA, and flow cytometry for only the chicken species. https://www.abcam.com/goat-chicken-igy-hl-alexa-fluor-568-ab175477.html

Eukaryotic cell lines

Policy information about cell lines

Cell line source(s)

1-hiPSCs, Rutgers University Cell and DNA Repository, Cat#: ND50028
2-hiPSC-derived dopaminergic neurons (iCell® Neurons), Cellular Dynamics International, Cat#: R1108
3- Human Astrocytes, ScienCell, Cat#:1800
4- Human Microglia, ATCC, Cat#: CRL-3304

	5- Human brain varcular pericytes, ScienCell, Cat#: 1200
Authentication	Validations for all cell sources are available on manufacturers' websites or associated literature:<br>1st Set of Lines:<br>1-Identity was confirmed by the manufacturer using SNP Trace. Pluripotency was confirmed by the manufacturer using Live colony image, AP Stain, Immunofluorscence, FACS, RUCDR Pluritest, iPSC Scorecard, Differentiation Scorecard. We confirmed genetic stability by karyotyping.<br>2-iCell DopaNeurons are comprised primarily of midbrain dopaminergic neurons as demonstrated by immunocytochemistry: microtube-associated protein 2 (MAP2) and LIM homeobox transcription factor 1 (Lmx1), day 7 post-plating; forkhead box protein A2 (FoxA2) and tyrosine hydroxylase (TH), day 14 post-plating; and FoxA2 and Lmx1, day 7 post-plating.<br>Genetic stability, pluripotency, and RFP expression were confirmed by the manufacturer.<br>3-Human Astrocytes (HA) from ScienCell Research Laboratories are isolated from human brain. HA are cryopreserved at passage one and delivered frozen. HA are characterized by immunofluorescence with antibody specific to GFAP. HA are negative for HIV-1, HBV, HCV, mycoplasma, bacteria, yeast, and fungi. HA are guaranteed to further expand for 10 population doublings under the conditions provided by ScienCell Research Laboratories.<br>4- Resting Migroglia cells were strongly positive for the microglia/macrophage marker IBA1, but negative for the astrocyte marker GFAP. Markers of activated microglia, namely MHCII and CD68 were negative in resting HMC3 cells, but upregulated after activation by IFN-gamma (10 ng/ml, 24 h).<br>5- Human Brain Vascular Pericytes (HBVP) from ScienCell Research Laboratories are isolated from human brain. HBVP are cryopreserved at passage one after purification and delivered frozen. HBVP are characterized by immunofluorescence with antibody specific to α-smooth muscle actin.
Mycoplasma contamination	Tested negative
Commonly misidentified lines<br>(See ICLAC register)	No commonly misidentified cell lines were used in the study
